# Excess mortality during the COVID-19 outbreak in Italy: a two-stage interrupted time-series analysis

**DOI:** 10.1093/ije/dyaa169

**Published:** 2020-10-14

**Authors:** Matteo Scortichini, Rochelle Schneider dos Santos, Francesca De’ Donato, Manuela De Sario, Paola Michelozzi, Marina Davoli, Pierre Masselot, Francesco Sera, Antonio Gasparrini

**Affiliations:** 1 Department of Public Health, Environments and Society, London School of Hygiene & Tropical Medicine (LSHTM), London, UK; 2 Department of Epidemiology, Lazio Regional Health Service, via Cristoforo Colombo, Rome, Italy; 3 Centre on Climate Change and Planetary Health, London School of Hygiene & Tropical Medicine (LSHTM), London, UK; 4 Centre for Statistical Methodology, London School of Hygiene & Tropical Medicine (LSHTM), London, UK

**Keywords:** SARS-CoV-2, COVID-19, epidemic, Italy, mortality, interrupted time series

## Abstract

**Background:**

Italy was the first country outside China to experience the impact of the COVID-19 pandemic, which resulted in a significant health burden. This study presents an analysis of the excess mortality across the 107 Italian provinces, stratified by sex, age group and period of the outbreak.

**Methods:**

The analysis was performed using a two-stage interrupted time-series design using daily mortality data for the period January 2015–May 2020. In the first stage, we performed province-level quasi-Poisson regression models, with smooth functions to define a baseline risk while accounting for trends and weather conditions and to flexibly estimate the variation in excess risk during the outbreak. Estimates were pooled in the second stage using a mixed-effects multivariate meta-analysis.

**Results:**

In the period 15 February–15 May 2020, we estimated an excess of 47 490 [95% empirical confidence intervals (eCIs): 43 984 to 50 362] deaths in Italy, corresponding to an increase of 29.5% (95% eCI: 26.8 to 31.9%) from the expected mortality. The analysis indicates a strong geographical pattern, with the majority of excess deaths occurring in northern regions, where few provinces experienced increases up to 800% during the peak in late March. There were differences by sex, age and area both in the overall impact and in its temporal distribution.

**Conclusion:**

This study offers a detailed picture of excess mortality during the first months of the COVID-19 pandemic in Italy. The strong geographical and temporal patterns can be related to the implementation of lockdown policies and multiple direct and indirect pathways in mortality risk.

**Key Messages**

- This study evaluated the mortality trends in Italy during the COVID-19 pandemic, reporting an excess of 47 490 [95% empirical confidence intervals (eCIs): 43 984 to 50 362] deaths in the period 15 February–15 May 2020, corresponding to an increase of 29.5% (95% eCI: 26.8 to 31.9%) from the expected mortality.
- There was a strong geographical pattern, with 71.0% of the estimated excess deaths occurring in just three northern regions (Lombardy, Veneto and Emilia-Romagna) and few provinces showing increases in mortality up to 800% during the peak of the pandemic.
- The impact was slightly higher in men compared with women, with 24 655 and 23 125 excess deaths, respectively. The risk varied by age, with the highest excess mortality in the group of people aged 70–79 years old and with a lower but measurable risk evident in those aged <60.
- The analysis by week suggests differential trends, with more delayed impacts in women and the elderly, and with the risk limited to a shorter period (March to mid-April) in Central and Southern Italy.
- These differences are likely related to the implementation of lockdown policies and to contributions from differential risk pathways, including deaths directly related to COVID-19 and indirect mortality due to other factors such as disruptions in the healthcare system.

## Introduction

The outbreak of the SARS-CoV-2 coronavirus originated in Wuhan, China, in December 2019 and quickly affected most countries worldwide in the first months of 2020. The infection causes COVID-19 disease—a severe acute respiratory syndrome that can lead to hospitalization and eventually death.^1^^,^^2^ As of 6 July 2020, almost 11.5 million cases of COVID-19 have been reported worldwide, resulting in 535 027 official deaths.^3^

Italy has been one of the worst-affected countries in the first months of the pandemic. The first case of SARS-CoV-2 infection not originating in China was reported on 20 February in the province of Lodi and the disease quickly spread across the north of the country.^4^ The number of reported cases with a validated diagnostic test rose to 1128 by the end of the month and peaked on 21 March with 6557 cases per day.^4^

A series of containment policies, both at national and local levels, have been implemented since the start of the outbreak. The Italian government declared the quarantine of 11 municipalities in Northern Italy on 21 February, which was then extended to the entire region of Lombardy on 8 March and finally to the whole country the next day. Strict lockdown measures were implemented thereafter, including social distancing, travel restrictions, closure of all non-essential businesses and industries and stay-at-home orders.^5^ Most of the strictest measures were not released until early May.

The pandemic put enormous pressure on the health system and it was particularly severe in Lombardy and a few other selected provinces in other northern regions, where hospitals were overwhelmed. Emergency and intensive-care units focused almost exclusively on COVID-19 patients, with admissions for other causes substantially reduced, whereas general-practice and outpatients activities were in many cases halted to prevent the transmission of the virus.^6^^,^^7^ The first fatality officially attributed to COVID-19 in Italy occurred in Veneto on 21 February. The number of deaths then started rising and, by 15 May, 31 610 deaths related to COVID-19 were reported.^4^

Previous assessment on the health impact focused on excess in all-cause mortality^8–10^—an indicator that offers a quantification of the overall burden of the disease, accounting for both direct and indirect effects.^11^ However, these analyses were limited to urban areas or used data aggregated by region, and they adopted relatively simple designs based on pre-post comparisons that do not account for temporal changes in risk. These can be due to seasonality or long-term trends in mortality and to yearly variation in risk factors such as weather. In particular, the mild winter of 2020 could have resulted in a lower mortality burden attributed to cold,^12^ therefore biasing downwards the quantification of excess mortality.

In this contribution, we performed an analysis of the excess mortality during the first months of the SARS-CoV-2 coronavirus outbreak across the 107 Italian provinces, stratified by sex, age group and period of the outbreak. The assessment is based on an official mortality data set released by the Italian Institute of Statistics (ISTAT) and it benefits from the application of a novel two-stage interrupted time-series design and flexible statistical methods to control for trends and variations risk factors. This study can therefore offer a comprehensive overview of the mortality impact of the COVID-19 pandemic in Italy.

## Methods

### Data

The analysis is based on the database released by ISTAT on 18 June 2020,^13^ which provides the number of daily deaths stratified by sex and age groups for 7904 municipalities in 107 provinces and 20 regions of Italy within the period 1 January 2015–15 May 2020 (Table 1). The database includes complete daily counts for 2015–2019 and early-release data for the 4.5 months of 2020 for 7270 municipalities (92% of the total) for which linkage with death registration was up to date. The mortality counts were aggregated by province in sex- and age-specific daily time series, and linked with daily mean-temperature data at the population-weighted centroid of the province from the ERA-5 reanalysis data set on the Copernicus climate data store,^14^ and weekly incidence of influenza cases at the regional level determined by laboratory tests.^15^

**Table 1:** Number of provinces, municipalities, coverage in the year 2020 and total deaths within the study period (1 January 2015–15 May 2020) by region and area of Italy

Region	Area	Provinces	Municipalities	Coverage (%)^a^	Deaths
Piemonte	North	8	1181	94.10	293 001
Valle d'Aosta	North	1	74	91.90	8084
Lombardia	North	12	1506	97.30	562 459
Trentino-Alto Adige	North	2	282	91.80	52 425
Veneto	North	7	563	89.90	268 053
Friuli-Venezia Giulia	North	4	215	93.50	79 719
Liguria	North	4	234	93.60	120 611
Emilia-Romagna	North	9	328	92.70	278 774
Toscana	Central	10	273	89.40	238 999
Umbria	Central	2	92	94.60	56 601
Marche	Central	5	228	89.50	96 889
Lazio	Central	5	378	82.50	314 502
Abruzzo	South	4	305	91.50	82 613
Molise	South	2	136	91.90	20 647
Campania	South	5	550	88.70	297 730
Puglia	South	6	257	91.10	214 697
Basilicata	South	2	131	93.10	34 653
Calabria	South	5	404	91.10	110 204
Sicilia	Islands	9	390	83.30	286 828
Sardegna	Islands	5	377	92.30	90 505
Italy		107	7904	92.00	3 507 994

a The coverage represents the percentage of municipalities by region with data available for the year 2020.

### Two-stage analysis

The statistical analysis was performed by applying a two-stage interrupted time-series model to quantify the time-varying excess risk for mortality during the COVID-19 outbreak compared with the pre-outbreak period, accounting for temporal trends and variation in other risk factors. In the first stage, at the province level, we applied a quasi-Poisson time-series regression model with smooth spline functions for time variables and observed predictors.^16^ Specifically, we included a linear term for time to model long-term trends, a cyclic cubic B-spline with three equally spaced knots for the day of the year to model seasonality, and indicators for day of the week to take into account weekly variations in mortality. The excess risk in mortality during the COVID-19 outbreak was defined through a constrained quadratic B-spline with four equally spaced knots for the days from 1 February 2020 to 15 May 2020 (outbreak period). This function constrains the excess risk to start from null at the beginning of February and then allows it to vary flexibly until the end of the study period. Potential differences in the underlying mortality risk due to non-optimal weather between the pre-outbreak and outbreak periods were controlled by including a term for the mean daily temperature. This was defined through a distributed lag non-linear model over 0–21 lag days,^17^ using a cross-basis parameterization defined in previous studies.^12^ It was decided not to control for influenza in the main model, as laboratory testing could be affected by the pandemic, thus resulting in an artificial reduction in flu incidence. This choice was tested in a sensitivity analysis, in which we controlled for the effect of influenza using a 2-week moving average of the reconstructed daily incidence.

The province-specific coefficients of the function defining the excess risk were then pooled in the second stage using a mixed-effects multivariate meta-analysis^18^ and the best linear unbiased predictions for each of the 107 provinces were extracted. This approach allows borrowing strengths in the estimates across provinces, while at the same time modelling flexible associations of multi-parameter functions.^19^

### Quantification of excess mortality

The model was performed on the province-specific aggregated series composed by the 7270 municipalities with full data and the estimates were used to compute the relative risk (RR) representing the excess in each province for each day of the outbreak period. We then reconstructed the complete mortality series for all the municipalities by using the data in the period 2015–2019 to compute, in each province, the proportion of deaths occurring in municipalities with full data and then applying the same proportion to the 4 months of 2020. The daily number of excess deaths was computed as (*RR* – 1)/RR*n, where *n* is the daily number of deaths, and aggregated by week during the outbreak period (from 1 February) and then in total for the period with reported COVID-19 mortality (from 15 February). The excess number and fraction of deaths were then computed for each province, region, area (North, Central, South and Islands) and for the whole of Italy. We computed empirical confidence intervals (eCIs) at 95% by Monte Carlo simulation of the coefficients of the quadratic B-spline modelling the excess risk, assuming a multivariate normal distribution using the point estimates and (co)variance matrix. Sex- and age-specific models were run separately to compute the excess across subgroups of the population.

Information on repositories, software and code for accessing the data and replicating the analysis are provided in the ‘Data Availability’ section below.

## Results

**Figure 1:**
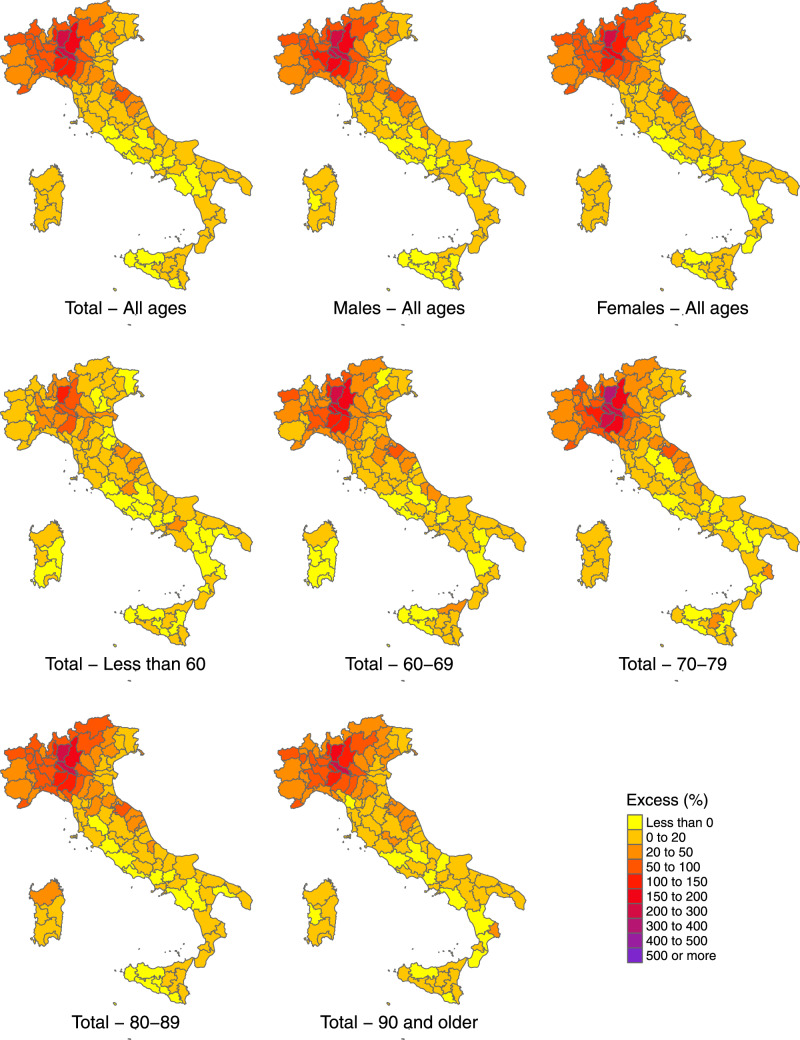
Maps of percentage excess in mortality during the intense period of the COVID-19 pandemic (15 February–15 May 2020) by province in Italy, in total and stratified by sex and age group

In the 3 months from 15 February to 15 May 2020, ∼208 320 deaths were registered in Italy, with an estimated increase of 47 490 (95% eCI: 43 984 to 50 362) from the expected baseline number when accounting for temporal trends and differences in temperature distribution (Table 2). This figure corresponds to a percentage excess of 29.5% (95% eCI: 26.8 to 31.9%). The excess shows a strong geographical pattern, with an extremely high rise in the number of deaths in the northern regions. Lombardy alone experienced a staggering 25 782 excess deaths and, together with two other northern regions (Veneto and Emilia-Romagna), accounts for 71.0% of all the excess mortality estimated in Italy. The maps in Figure 1 provide a better representation of the geographical distribution, showing a percentage increase in deaths that reaches almost 400% in a few selected northern provinces. The excess mortality is comparatively milder in Central Italy and considerably smaller or absent in the South and the Islands, although with a patchy distribution and isolated spots such as in the provinces of Pesaro-Urbino (East coast) and Crotone (the ‘bottom of the foot’ in the South).

**Table 2:** Number of total deaths and estimated excess (with 95% empirical confidence intervals) during the intense period of the COVID-19 pandemic (15 February–15 May 2020) in Italy, by sex

	Both sexes		Males		Females	
	Total	Excess	Total	Excess	Total	Excess
Piemonte	18 417	5577 (5095 to 6004)	8860	2756 (2577 to 2898)	9557	2810 (2543 to 3046)
Valle d'Aosta	541	194 (159 to 224)	253	85 (65 to 103)	289	111 (87 to 129)
Lombardia	50 874	25 782 (24 806 to 26 703)	25 178	13 320 (12 844 to 13 673)	25 697	12 447 (12 098 to 12 731)
Trentino-Alto Adige	3688	1227 (1057 to 1384)	1700	518 (494 to 533)	1987	709 (613 to 787)
Veneto	14 905	2317 (1793 to 2776)	7120	1146 (985 to 1263)	7785	1185 (727 to 1582)
Friuli-Venezia Giulia	4135	461 (299 to 601)	1947	243 (147 to 310)	2188	213 (105 to 308)
Liguria	7599	2346 (2072 to 2595)	3570	1099 (1039 to 1146)	4029	1249 (1168 to 1309)
Emilia-Romagna	18 249	5605 (5212 to 5938)	8906	3020 (2858 to 3122)	9343	2573 (2333 to 2784)
Toscana	12 210	1416 (1137 to 1643)	5897	794 (493 to 1037)	6313	633 (450 to 772)
Umbria	2651	83 (–51 to 200)	1233	15 (–81 to 104)	1418	80 (–22 to 170)
Marche	5776	1415 (1283 to 1523)	2852	817 (769 to 842)	2924	612 (473 to 715)
Lazio	13 972	–548 (–1032 to –97)	6786	–154 (–421 to 65)	7187	–325 (–646 to –33)
Abruzzo	4162	419 (181 to 644)	2062	212 (135 to 277)	2101	225 (99 to 322)
Molise	931	23 (–42 to 80)	442	3 (–56 to 48)	489	31 (–15 to 66)
Campania	13 816	125 (–334 to 524)	6937	257 (–176 to 655)	6879	–98 (–377 to 132)
Puglia	10 939	685 (414 to 885)	5406	363 (75 to 602)	5533	322 (66 to 527)
Basilicata	1715	–6 (–126 to 86)	805	–28 (–100 to 25)	911	34 (–47 to 104)
Calabria	5396	119 (–40 to 244)	2660	83 (–101 to 244)	2736	53 (–131 to 209)
Sicilia	13 742	11 (–564 to 487)	6672	24 (–271 to 258)	7069	70 (–179 to 252)
Sardegna	4602	238 (53 to 407)	2282	82 (–68 to 210)	2320	190 (–9 to 344)
Italy	208 320	47 490 (43 984 to 50 362)	101 568	24 655 (22 604 to 26 215)	106 754	23 125 (20 997 to 24 609)

**Figure 2:**
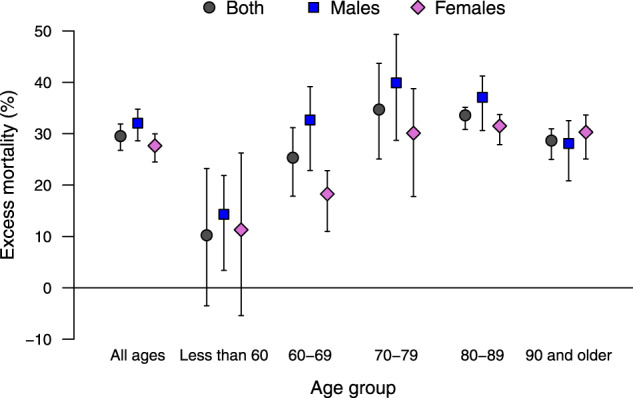
Percentage excess in mortality (with 95% empirical confidence intervals) during the intense period of the COVID-19 pandemic (15 February–15 May 2020) in Italy, in total and stratified by sex and age group

The increase in mortality is slightly higher in men compared with women, with a total of 24 655 and 23 125, corresponding to percentage increases of 32.1% (95% eCI: 28.6 to 34.8%) and 27.7% (95% eCI: 24.5 to 30.0%), respectively (Table 2). Figure 2 reports the results stratified by age and sex, indicating a lower excess mortality in women in the age groups between 60 and 89 years old but almost identical to males in younger and older people. Excess mortality was substantially lower in people <60 years old, although this group still shows a percentage excess of 10.2% (95% eCI: –3.5 to 23.2%) in the outbreak period. The maps in Figure 1 suggest a similar geographical distribution of the excess deaths across sex and age groups.

**Figure 3:**
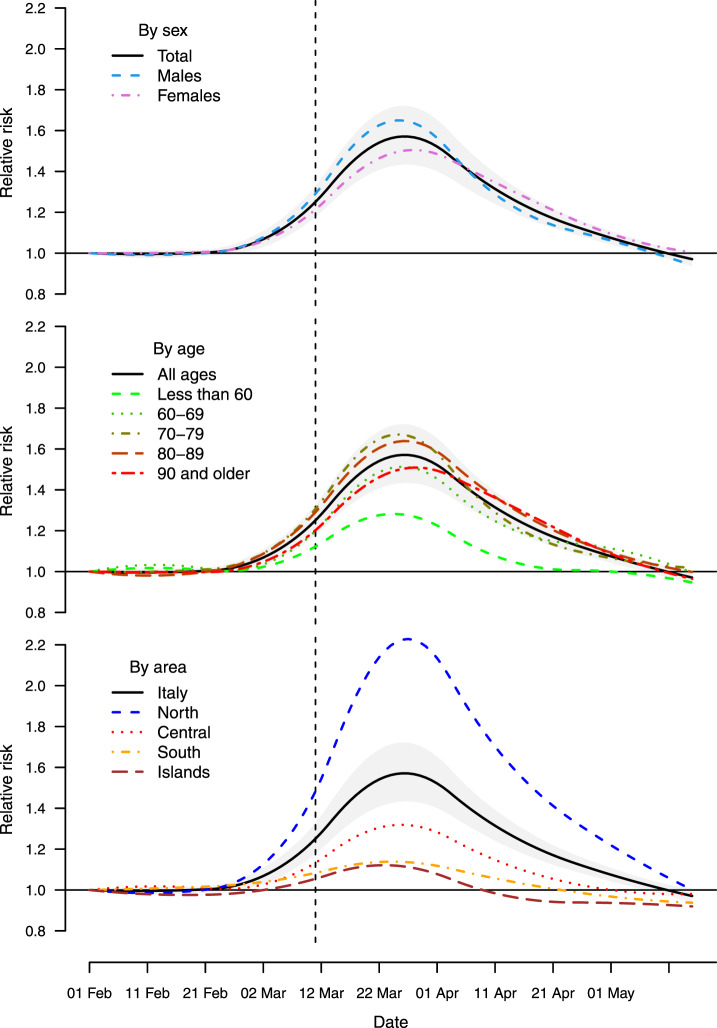
Trend in excess risk (relative risk, RR) during the period 1 February–15 May 2020 in Italy by sex, age and geographical area compared with the total (with a band corresponding to the 95% empirical confidence intervals)

Figure 3 shows the trend in RR of mortality during the SARS-CoV-2 outbreak period (1 February–15 May) by sex, age group and area. The graphs suggest that the risk started increasing above the baseline at the beginning of March and that the peak was reached at the end of the same month, almost 20 days after the implementation of lockdown measures. Given the uneven geographical distribution of the risk, with the densely populated provinces of the North mostly affected, this increased risks translates into a larger total excess in mortality, which reached 78.7% (95% eCI: 70.6 to 85.5%) above the baseline in the week of 18–24 March (figures reported in the Supplementary Data, available as Supplementary data at *IJE* online). The risk then decreased during April, with evidence of an excess mortality until at least the first week of May. The temporal distribution of risk is similar across sex and age groups (top and middle panels), with an indication of a slight delayed peak for women and very elderly (90 years old and older). The RR for the <60 age group reaches a lower peak just above 1.2 and it returns to the baseline level by mid-April. Consistently with what has been discussed above, the analysis by geographical area (bottom panel) suggests a very high mortality risk in the North that across the region is more than twice at the peak (RR above 2.0), whereas other areas show milder increases and earlier peaks. Some provinces showed staggering increases, with the percentage excess in Bergamo reaching 858.7% (95% eCI: 771.9 to 969.5%) in the week of 18–24 March (see Supplementary Data, available as Supplementary data at *IJE* online). There is some indication of an RR <1 in the late stage of the epidemics in Central and Southern Italy, which can suggest a ‘harvesting effect’, whereby some of the deaths are only anticipated by a few weeks.^20^ This phenomenon can be explored when data for a longer time period become available.

Controlling for the additional effect of influenza has little impact, with an estimated excess of 48 569 compared with 47 490 in the main model.

The full set of results, including the number and fraction of excess deaths (with 95% eCI) by geographical aggregation (provinces, region and full country), sex, age groups and period (15 February–15 May 2020, and then by week starting from 1 February), is provided in the Supplementary Data, available as Supplementary data at *IJE* online.

## Conclusion

This study offers a detailed assessment of the mortality for all causes in Italy during the COVID-19 pandemic wave in the first months of 2020. The estimate of an excess of 47 490 deaths is far higher than the number of 31 610 deaths officially recorded as related to COVID-19 in the same months. The increased mortality risk shows a strong geographical pattern, with a few northern provinces carrying the majority of excess deaths. Stratified analyses indicate differences in the impact by sex and age, whereas the analysis of the trend shows that excess mortality peaked at the end of March.

The most striking result of the analysis is the strong north-to-south geographical gradient in the impact of COVID-19 in Italy, consistent with previous studies from Italy.^8–10^ The vast majority of excess deaths occurred in selected northern provinces. However, differently from what was previously reported,^10^ this study is able to identify significant increases in many provinces and regions of the South as well. The reasons for such North–South differences are still debated. A role was likely being played by the timing and characteristics of the SARS-CoV-2 outbreak, which started in the provinces of the North with established trade connections with China and was amplified by the higher population density and internal travel.^21^ The implementation of strict lockdown policies, including the restriction in interregional movements, has likely contributed to reducing the spread of the infection and the consequent excess mortality in other regions, particularly in the South. This aspect can also explain the patchy map of risk across the country, where hotspots of risk can be the result of the influx of infections from high-prevalence provinces of the North before strict lockdown policies where implemented. Other suggested reasons for the geographical distribution of excess mortality are the occurrence of mass-gathering events that boosted local numbers of infections^22^ and regional differences in health-service organization, socio-economic determinants and urbanization.^23–25^

The assessment of results of the stratified analysis offers interesting clues on sex and age differentials. Whereas this study confirms the higher risk in men, the difference is comparatively small, with a percentage increase of 32.1% vs 27.7% in women. The analysis by age in Figure 2 reveals that the highest excess occurred among the 70–79 year-olds, but was lower in the younger and older groups. The risk is even lower but still present in the rest of the population, with evidence of excess deaths even in people <60 years old. The graph in Figure 3 shows instead some differences in the timing of the excess mortality across sex, age groups and area. The more delayed peak in women and the very elderly can be indicative of differential risk pathways, for instance related to the reduction in access to emergency services during the lockdown or the delayed surge of infections in nursing care homes. The early decrease and then below-average mortality in Central and Southern Italy can instead imply that some of the excess is due to anticipation of death in frail individuals. These suggestive results can form the basis of future analyses.

The figures reported in this study can be compared with those previously published in peer-reviewed articles, although the comparison is made difficult by the different study population, study period, data-collection procedures and analytical methods. Michelozzi and colleagues analysed data collected within a surveillance system from 19 Italian cities, reporting a total excess mortality of 45% from the beginning of March until 18 April 2020.^9^ The increase was higher in northern cities, in men and in the elderly, although with estimates that are somewhat different from the present analysis, in part probably due to the focus on urban areas and the different period. Magnani and colleagues used a previous release of the data by ISTAT on 4433 municipalities covering the whole Italian territory. They extrapolated the excess to the total Italian population, reporting an expected excess of 44 352.5 deaths in the group of people >60 years old and 680.4 deaths in younger people, between 1 March and 15 April.^10^ They, however, reported negative estimates in several regions, in particular for the younger group. These differences can be due to the simple pre-post comparison adopted in these studies, which does not account for temporal trends and for the milder weather conditions in 2020.

This study benefits from the application of an advanced two-stage design and statistical models that allow a flexible estimation of the excess mortality risk and a definition of the baseline risk that considers temporal trends and variations in known risk factors. The analysis is performed at the province level and stratified by sex, age and week periods, thus offering a fine characterization of the impact of the COVID-19 pandemic in Italy. Data and code are available, making the analysis entirely reproducible, and the full set of results is shown in the appendix and through a web application. Some limitations must, however, be acknowledged. First, the low number of cases prevents the full application of the two-stage modelling in age groups <50 years old and therefore the ability to address the question about to what extent COVID-19 affects young people. Furthermore, the interrupted time-series design defines a simple pre-post comparison that cannot provide information about the various mechanisms leading to increased risks. The question about controlling or not for the incidence of seasonal influenza is emblematic with respect to this problem. Similarly, whereas the analysis of all-cause mortality can provide a good estimate of the overall burden, it offers no information about the various mechanisms, patho-physiological or of different nature, leading to increased deaths. Finally, the analysis is based on an early-release data set from ISTAT that has not been consolidated and this may lead to an underestimation of the number of deaths that occurred in 2020 and therefore of the excess during the pandemic. These issues can be addressed when additional data and information on the COVID-19 pandemic are released.

In conclusion, this study offers a detailed picture of excess mortality during the first months of the COVID-19 pandemic in Italy. The analysis indicates that the excess mortality during the outbreak has been far higher than the number of deaths officially reported as related to COVID-19, with strong geographical differences and patterns of risk that vary by sex, age and period. The figures can be used in future research to explain differential risks across regions and subgroups, and the role of national or local policies implemented during the period.

## Supplementary data

Supplementary data are available at *IJE* online.

## Funding

This work was supported by the Medical Research Council-UK (Grant ID: MR/M022625/1) and the European Union’s Horizon 2020 Project Exhaustion (Grant ID: 820655). 

## Supplementary Material

dyaa169_Supplementary_Data

## References

[1] Onder G, Rezza G, Brusaferro S. Case-fatality rate and characteristics of patients dying in relation to COVID-19 in Italy. JAMA 2020;323:1775–6. 32203977 10.1001/jama.2020.4683

[2] Guan W, Ni Z, Hu Y et al Clinical characteristics of coronavirus disease 2019 in China. N Engl J Med 2020;382:1708–20. 32109013 10.1056/NEJMoa2002032 PMC7092819

[3] John Hopkins University. Covid-19 dashboard. https://coronavirus.jhu.edu/map.html (26 June 2020, date last accessed).

[4] Department for Civil Protection. [COVID-19 Italy: regional data digital repository]. 2020 https://github.com/pcm-dpc/COVID-19/tree/master/dati-regioni (26 June 2020, date last accessed).

[5] Italian Government—Presidency of the Council of Ministers. #IoRestoaCasa, measures for containment and management of the COVID-19 epidemiological emergency. http://www.governo.it/it/iorestoacasa-misure-governo (26 June 2020, date last accessed).

[6] Fagiuoli S, Lorini FL, Remuzzi G. Adaptations and lessons in the province of Bergamo. N Engl J Med 2020;382:e71. 32369276 10.1056/NEJMc2011599 PMC7219535

[7] Grasselli G, Pesenti A, Cecconi M. Critical care utilization for the COVID-19 outbreak in Lombardy, Italy: early experience and forecast during an emergency response. JAMA 2020;323:1545. 32167538 10.1001/jama.2020.4031

[8] Davoli M, Andamento della mortalità giornaliera (SiSMG) nelle città italiane in relazione all’epidemia di Covid-19. Report 1 Febbraio–12 Maggio 2020 (Rapporto FINALE). http://www.deplazio.net/images/stories/SISMG/SISMG_COVID19.pdf? fbclid=IwAR1jjtKJaGj4yBgB5DuDTsHND1u7sllvuM3C5ZnOL 40WR65nxqlZx6e2bdQ (26 June 2020, date last accessed).

[9] Michelozzi P, de’Donato F, Scortichini M et al Mortality impacts of the coronavirus disease (COVID-19) outbreak by sex and age: rapid mortality surveillance system, Italy, 1 February to 18 April 2020. Euro Surveillaince 2020;25:2000620. 10.2807/1560-7917.ES.2020.25.19.2000620 PMC7238743 32431289

[10] Magnani C, Azzolina D, Gallo E et al How large was the mortality increase directly and indirectly caused by the COVID-19 epidemic? An analysis on all-causes mortality data in Italy. Int J Environ Res Public Health 2020;17:3452. 10.3390/ijerph17103452 PMC7277828 32429172

[11] Zylke JW, Bauchner H. Mortality and morbidity: the measure of a pandemic. JAMA 2020;324:458. 32609308 10.1001/jama.2020.11761

[12] Gasparrini A, Guo Y, Hashizume M et al Mortality risk attributable to high and low ambient temperature: a multicountry observational study. Lancet 2015;386:369–75. 26003380 10.1016/S0140-6736(14)62114-0 PMC4521077

[13] Dati di mortalità: cosa produce l’Istat. https://www.istat.it/it/archivio/240401 (16 June 2020, date last accessed).

[14] ECMWF. ERA5 hourly data on single levels from 1979 to present. https://cds.climate.copernicus.eu/cdsapp#!/dataset/reanalysis-era5-single-levels? tab=form (12 June 2020, date last accessed).

[15] Influnet. Italian Surveillance Influenza network. Ministero della Salute 2020 http://www.salute.gov.it/portale/influenza/homeInfluenza.jsp (9 June 2020, date last accessed).

[16] Bhaskaran K, Gasparrini A, Hajat S, Smeeth L, Armstrong B. Time series regression studies in environmental epidemiology. Int J Epidemiol 2013;42:1187–95. 23760528 10.1093/ije/dyt092 PMC3780998

[17] Gasparrini A. Modeling exposure-lag-response associations with distributed lag non-linear models. Stat Med 2014;33:881–99. 24027094 10.1002/sim.5963 PMC4098103

[18] Sera F, Armstrong B, Blangiardo M, Gasparrini A. An extended mixed-effects framework for meta-analysis. Stat Med 2019;38:5429–44. 31647135 10.1002/sim.8362

[19] Gasparrini A, Armstrong B, Kenward MG. Multivariate meta-analysis for non-linear and other multi-parameter associations. Stat Med 2012;31:3821–39. 22807043 10.1002/sim.5471 PMC3546395

[20] Rabl A. Air pollution mortality: harvesting and loss of life expectancy. J Toxicol Environ Health A 2005;68:1175–80. 16024496 10.1080/15287390590936049

[21] Rudan I. A cascade of causes that led to the COVID-19 tragedy in Italy and in other European Union countries. J Glob Health 2020;10:010335. 32257150 10.7189/jogh-10-010335 PMC7125421

[22] Nuana D, Brassey J. *What Is the Evidence for Mass Gatherings during Global Pandemics?* Oxford Covid-19 Evidence Service, 2020.

[23] Piccininni M, Rohmann JL, Foresti L et al Use of all cause mortality to quantify the consequences of covid-19 in Nembro, Lombardy: descriptive study. BMJ 2020;369:m1835. 32409488 10.1136/bmj.m1835 PMC7223479

[24] Boccia S, Ricciardi W, Ioannidis JPA. What other countries can learn from Italy during the COVID-19 pandemic. JAMA Intern Med 2020;180:927. 32259190 10.1001/jamainternmed.2020.1447

[25] Piscitelli P, Miani A, Mazza A et al Health-care inequalities in Italy: challenges for the Government. Lancet Public Health 2019;4:e605. 31812237 10.1016/S2468-2667(19)30229-4

